# Effects of train vibration load on the structure and hydraulic properties of soils

**DOI:** 10.1038/s41598-024-57956-5

**Published:** 2024-03-28

**Authors:** Kai Han, Jiading Wang, Tao Xiao, Shan Li, Dengfei Zhang, Haoyu Dong

**Affiliations:** State Key Laboratory of Continental Dynamics, Department of Geology, Northwest University, Xi’an, 710069 China

**Keywords:** Train vibration, Soil–water characteristic, Hydraulic properties, Soil structure, Fractal dimension, Civil engineering, Hydrology, Geology, Hydrogeology

## Abstract

Investigating the impact of train-induced vibration loads on soil hydraulic properties, this study conducted experiments using a self-designed indoor soil seepage platform that incorporates vibration loads. The experiments were complemented with scanning electron microscopy to analyze the influence of train-induced vibration loads on soil hydraulic conductivity and its evolutionary characteristics under different vibration frequencies. The experimental results indicated that as the vibration frequency increases from no vibration (0 Hz) to 20 Hz, the time required for the soil volumetric moisture content to reach its peak and stabilize decreases rapidly. However, after the vibration frequency exceeds 20 Hz, the rate at which the time required for the volumetric moisture content to reach its peak and stabilize decreases slows down. Furthermore, the soil pore water pressure increases with the increase in vibration frequency. At a vibration frequency of 80 Hz, the peak value of pore water pressure increases by 105% compared to the non-vibration state, suggesting that higher vibration frequencies promote the development and acceleration of soil pore moisture migration. Additionally, as the vibration frequency increases, the soil hydraulic conductivity initially experiences a rapid increase, with a growth rate ranging from 40.1 to 47.4%. However, after the frequency exceeds 20 Hz, this growth rate significantly decreases, settling to only 18.6% to 7.8%. When the soil was subjected to a vibration load, the scanning electron microscopy test revealed alterations in its pore structure. Micropores and small pores transformed into macropores and mesopores. Additionally, the microstructural parameters indicated that vibration load decreased the complexity of soil pores, thereby speeding up the hydraulic conduction process. This, in turn, affected the hydraulic properties of the soil and established a relationship between pore structure complexity and soil hydraulic properties.

## Introduction

Recentlly, the frequency of engineering construction activities, particularly infrastructure construction, has increased in northwest China^1–3^. As a result, it has become crucial to understand soil behavior (i.e., mechnical behavior and hydraulic behavior) under mechanical loads, such as train vibrations (Lazorenko et al.^4,5^). Although the intensity of these dynamic loads is lower than that of earthquakes, long-term, and repeated dynamic loading can still cause soil loosening and cracking, which in turn affects hydraulic properties and microstructure of soils (such as pore area ratio, pore fractal dimension, aggregate particle size, and packing density)^6,7^. The hydraulic properties and microstructure of soil not only determine the movement of water in the soil but also influence the interaction between surface water and groundwater. This interaction plays a decisive role in geological disasters, engineering construction, and environmental changes^8–10^. The behavior of soil under dynamic loads, such as traffic vibration, exhibits significant complexity and variability^11^. For instance, train vibrations can increase the number of large pores, alter the soil structure, affect the hydraulic properties of the soil, and even result in slope sliding or landslides, posing a threat to railway safety^12^. Therefore, studying the soil's hydraulic properties and microstructure under the influence of train vibrations holds great significance for various engineering applications.

Most current research on soil hydraulic properties primarily focuses on investigating the influence of internal factors (such as soil structure^13^, initial dry density, and humidity^14^) as well as external environmental factors (e.g., dry–wet cycles^15^,An et al.^16^, freeze–thaw cycles^17,18^, temperature, and stress^19,20^) on hydraulic properties. Moreover, studies have also been conducted on the development of corresponding hydraulic conductivity functions. Additionally, mineral dissolution, cation exchange, and adsorption contribute to the dissolution and diffusion of cementing materials, which enhance the connectivity or crack development of closed pores, ultimately increasing the soil's hydraulic conductivity^21^. Meanwhile, one-dimensional soil column model tests^22,23^ and seepage numerical simulations^24,25^, which consider anisotropy and self-gravity stress, have been extensively utilized to examine the effects of rainfall intensity, grain size distribution, dry density, and initial moisture content on infilration process in the soil. These studies have led to the discovery of the impact of rainfall intensity and the advancement of wetting fronts^26,27^, as well as the identification of the mechanism by which gas phase obstruction hinders infiltration^28–30^. Furthermore, it has been emphasized that the macroscopic mechanical and hydraulic properties of soil are governed by its microstructure^31–33^. Hence, gaining a profound understanding of changes in soil microstructure holds paramount importance in comprehensively comprehending soil hydraulic properties.

Microstructural changes resulting from natural and anthropogenic factors, such as dry–wet cycles and freeze–thaw cycles, significantly impact the hydraulic properties of soils^34–36^. Additionally, soil structure undergoes seasonal expansion and contraction, leading to the variation of the pore-size distribution and grain size distribution^37^. Various experimental techniques, such as mercury intrusion porosimetry, optical microscopy, gas adsorption, X-ray micro-computed tomography (micro-CT), have been employed to characterize the microstructure and parameters of soil^38–40^. Among these techniques, scanning electron microscopy (SEM) has been widely used due to its cost-effectiveness and efficiency. SEM has proven valuable in studying the pore structure of soil.^31,31,39^) provided a comprehensive understanding of the microstructural evolution of cohesive soils during wetting and drying cycles. Prakongkep et al.^41^ utilized SEM in combination with image analysis methods to characterize the size and shape of sand particles in soil. Their analysis further revealed the relationship between these characteristics and the geological origin and depositional history of the soil. Eisenhauer et al.^42^ emphasized the application of SEM in soil ecological research, particularly its potential to explore complex interactions and causal relationships within soil ecosystems. There is little research on the impact of locomotive vibration on soil microstructure.

The objective of this study is to examine the hydraulic properties and changes in microstructure of soil subjected to vibration loading. To accomplish this, we utilized a specially designed seepage device to determine the hydraulic characteristics of the soil under different moisture levels and vibration frequencies. Subsequently, the soil underwent SEM tests to analyze the changes in microscopic parameters such as pore area proportions and pore fractal dimensions, while considering different initial moisture contents and vibration frequencies. Additionally, the correlation between the fractal dimensions of soil pores and hydraulic conductivity was explored. Lastly, the study provided a microscopic perspective to discuss the relationship between soil pore structure and hydraulic properties.

## Materials and methods

### Sample collection and preparation

**Figure 1:**
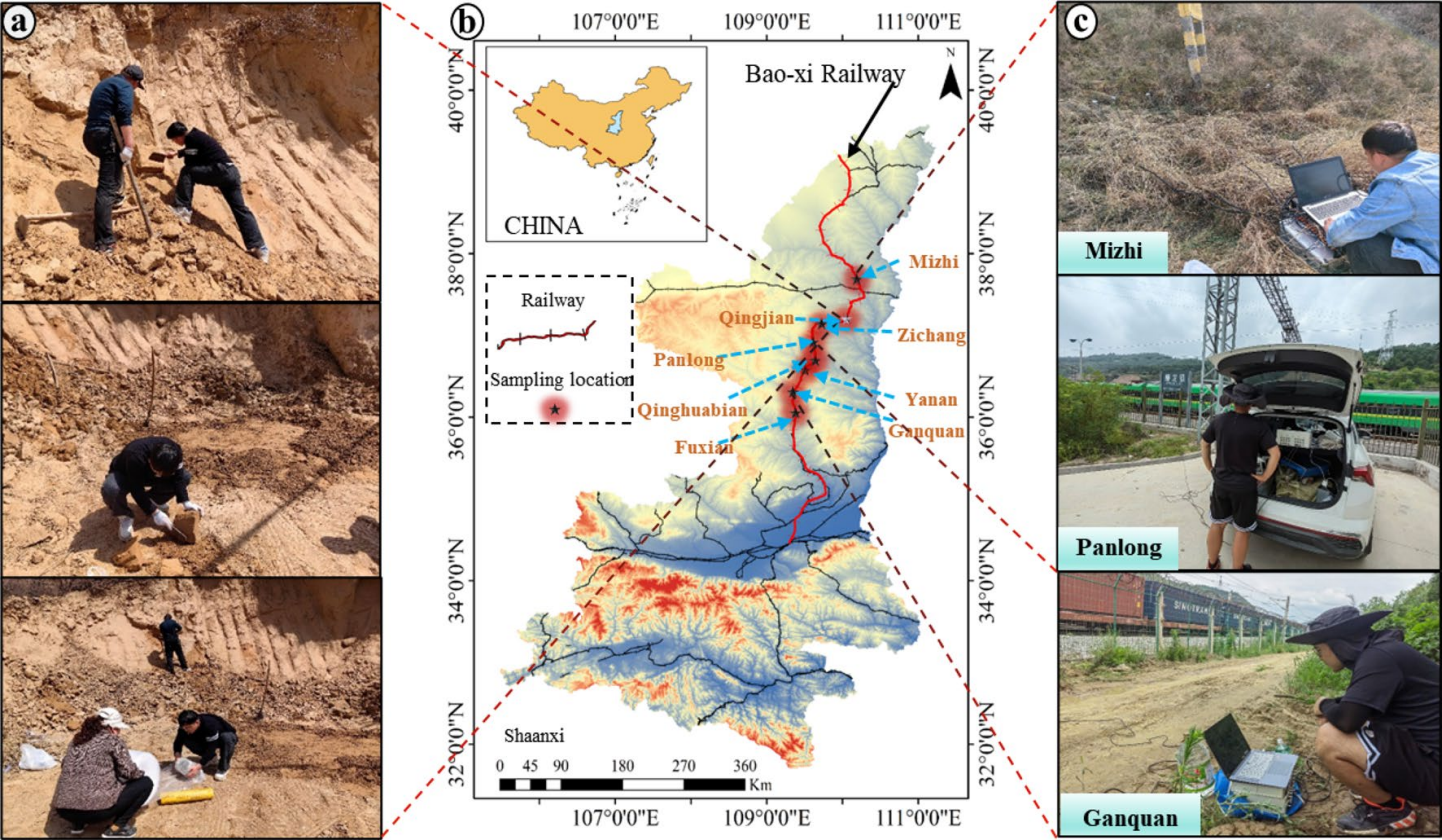
Sampling and monitoring locations, Baoxi Railway, Shaanxi Province, China.

This study conducted research on the Baoxi Railway in Shaanxi Province. Eight representative areas were selected for field surveys and soil sampling, while also monitoring acceleration parameters (see Fig. 1). The effects of vibration loading on the soil structure and hydraulic properties were investigated using Malan loess from a railway slope in Qingjian County, Shaanxi Province. The soil material was obtained from a depth of 3 m below the ground surface. Once sampled, the loess was carefully sealed in a bag and transported back to the laboratory. At the laboratory, the loess was allowed to naturally dry and then crushed in preparation for further tests.

**Figure 2:**
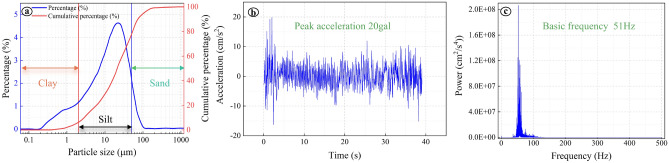
Field monitoring data. (**a**) Particle size distribution characteristics of soil samples; (**b**) On-site acceleration time history curve; (**c**) Spectrum analysis of trains.

Figure 2a presents the particle size distribution curve of the soil sample being tested. It can be observed that the loess soil tested has a high sand content, which has the potential to impact the hydraulic conductivity characteristics of the soil when subjected to vibration. Figure 2b displays the acceleration time history curve of the soil under the influence of train vibration, as obtained from field monitoring. Analysis of this curve indicates that the peak acceleration generated by train vibration measures at 20 gal. Figure 2c showcases the acceleration time history curve after Fourier transformation. The power spectral density diagram illustrates that the main frequency component is 51 Hz, which corresponds to the primary frequency component of the vibration signal. Research conducted by Wang et al.^12^ indicates that vibration loads with frequencies ranging from 0 to 20 Hz can accelerate seepage. However, it is important to note that the variation in the main frequency of train vibration across different regions is reasonable, given factors such as monitoring location and train type.

Table 1 presents the measurements of the soil samples' physical characteristics, conducted in accordance with the national standard GB/T50123-2019.

**Table 1:** Basic physical properties of Qing Jian Loess.

*w*_*n*_ (%)	*ρ*_*d*_ (g/cm^3^)	*w*_*L*_ (%)	*w*_*p*_ (%)	*I*_*P*_ (%)	Silt (%)	Clay (%)	Sand (%)
12.8	1.32	26.30	16.20	12.41	66.6	10.2	23.2

### Saturated seepage test

#### Saturated seepage test design

**Figure 3:**
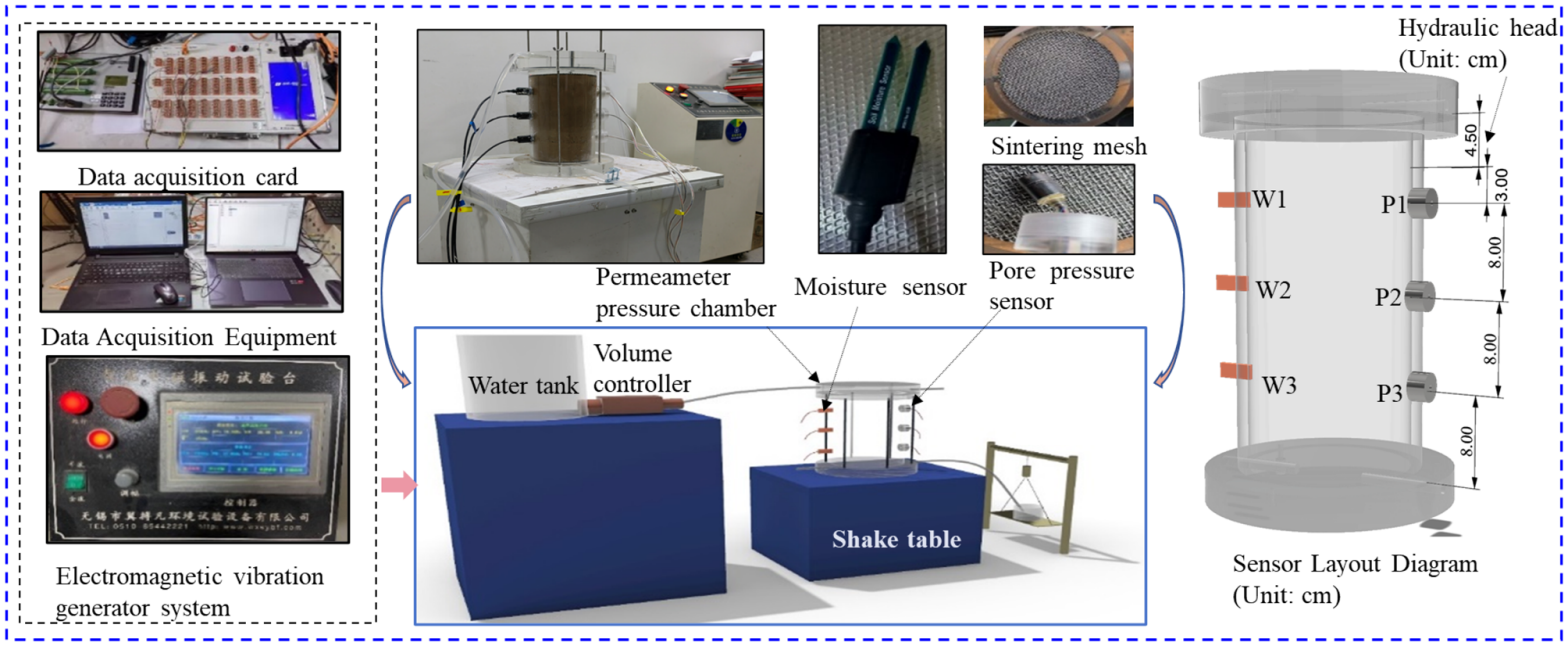
Test device for saturated permeability under vibration load.

To examine the influence of vibration load on soil hydraulic properties, a custom indoor seepage test platform was employed in this study. Figure 3 provides an illustration of this experimental setup. Seepage tests serve as a common approach for assessing saturated hydraulic conductivity (*K*_*s*_), representing the ease of water flow through porous materials^43^,Tang et al.^31,39^. In this investigation, we conducted measurements utilizing the constant head test method, a widely accepted technique for evaluating the permeability of saturated soils^44,45^.

Plexiglass tubes with an outer diameter of 160 mm, an inner diameter of 150 mm, and a length of 300 mm were used to construct 1D soil columns. These columns were designed to support soil compaction while maintaining a consistent water head. Advanced sensors were integrated into the setup for real-time data collection, and an electromagnetic pump was included to regulate flow rate and water pressure. A gap was intentionally left between the top of the soil column model and the upper cover of the plexiglass model to maintain a constant hydraulic head (4.5 cm).

In this experiment, soil moisture sensors (model MTD05), named W1, W2, and W3, along with pore water pressure sensors (model BWMK), were strategically installed at various depths within the soil column to accurately monitor the complex dynamics of soil moisture and pore water pressure (PWP). Specifically, the moisture sensors were positioned at distances of 3 cm (W1), 11 cm (W2), and 19 cm (W3) from the top of the column. This layout was chosen to facilitate a detailed examination of the vertical moisture gradient, enabling precise monitoring of moisture variations across different depths—a key aspect in assessing the soil's hydraulic properties under varying conditions.

Complementing the moisture data, pore pressure sensors labeled P1, P2, and P3 were placed at depths that correspond to the locations of moisture sensors W1, W2, and W3, respectively. This alignment allows for a direct comparison between moisture content and pore pressure data at identical vertical locations, offering an integrated profile of the soil's hydrological behavior. Sensor calibration was meticulously carried out following the guidelines provided in the MTD05 manual, ensuring the accuracy and reliability of the measurements. The selection of these high-resolution sensors was critical for detecting subtle changes in soil conditions, essential for an in-depth investigation of soil moisture dynamics under the test conditions presented.

To address potential issues of soil dispersion and maintain the integrity of the experimental setup, sintered nets with a specified mesh size were placed at both ends of the soil column. These nets allow for the unobstructed movement of water while preventing soil displacement, thus ensuring the consistency and reliability of experimental results across multiple tests.

#### Saturated seepage test procedure

Conducting research on the acceleration saturation effect of locomotive vibration involved collecting acceleration response analysis data on-site. An electromagnetic vibration table was then used to apply an equivalent locomotive vibration load. Experimental conditions included a sine wave with an applied amplitude (A) of 0.3 mm. The frequency (*f*) varied across vibration loads of 0, 20, 40, 60, and 80 Hz. Moreover, the initial moisture content (*w*_*0*_) varied under the same vibration loads. For additional details on the experimental control conditions, please consult Table 2.

**Table 2:** Saturated hydraulic conductivity tests under vibration.

Test number	*A* (mm)	*w*_0_ (%)	*f* (Hz)
T-1–1	0.3	5.0	0
T-1–2			20
T-1–3			40
T-1–4			60
T-1–5			80
T-2–1		10.0	0
T-2–2			20
T-2–3			40
T-2–4			60
T-2–5			80
T-3–1		15.0	0
T-3–2			20
T-3–3			40
T-3–4			60
T-3–5			80
T-4–1		20.0	0
T-4–2			20
T-4–3			40
T-4–4			60
T-4–5			80

During the test, both vibration load and constant water head were simultaneously applied. The PWP sensor was utilized to collect the PWP time history curves at various heights of the soil column in real-time. In addition, the soil moisture sensor was used to record real-time volumetric moisture content time history curves at various heights of the soil column. Throughout the test, the variation in wetting peak depth was monitored, the duration of stable seepage was recorded, the water outflow time at the bottom of the soil column was documented, and a pressure sensor was utilized to measure the amount of water extracted. The saturated hydraulic conductivity of the soil was then calculated using formula (1).

1 $$k = \frac{Q*l}{{A*h*t}}$$

where* k* represents the saturated hydraulic conductivity. *Q* denotes the water volume that traverses the soil sample within the time frame of *t*. *l* signifies the length of the soil specimen. *A* represents the cross-sectional area of the soil sample. *h* signifies the discrepancy in hydraulic head. *t* refers to the duration needed for water to traverse the soil specimen.

### SEM test

#### SEM test procedures

To conduct microstructure testing on the soil samples that underwent the seepage test, the samples were initially dried using vacuum freeze-drying equipment. Subsequently, a shallow cut was made in the middle of the long side of the loess strip, dividing it into two small cubes. The suspended particles on the fresh side of the cube were removed using an ear-cleaning ball, and the sample was affixed to the sample holder using conductive adhesive. The surface was then metal sprayed using an ion sputtering device. For observation under the FEI Quanta 400 scanning electron microscope, a high voltage of 20 kV was set. Additional, considering the findings of previous research^46–48^, the magnification of microscopic images was set at 500 times and 1000 times, with a minimum of 6 images taken for each sample.

#### SEM image processing and analysis

The images obtained from the electron microscope were processed using Pythone software. Initially, the scanning image of the sample was converted into a grayscale format, followed by binarization based on the grayscale values to differentiate the ruler and the background. Morphological operations were then employed to eliminate noise smaller than 500 pixels, thereby enhancing the quality of the image. Subsequently, the connected region labeling method was utilized to identify and mark the pores in the binary image. By analyzing the characteristics of the connected regions, the length of the ruler was determined, enabling the calculation of the actual length corresponding to each pixel (micron/pixel)^49,50^. Based on this information, the size of soil pores was analyzed and classified according to their equivalent diameter.

To demonstrate the classification results, we assigned different colors to pores based on their diameters. Pores with diameters less than 2 microns were marked as red (micropores), pores with diameters between 2 and 8 microns were marked as green (small pores), and pores with diameters between 8 and 32 microns were marked as blue (mesopores). Additionally, pores with diameters between 32 and 200 microns were marked as yellow (macropores). To enhance clarity and highlight the distribution of pores of different sizes, we created a new image with an all-white background and overlaid it with the colored pores.

## Results and analysis

### Effect of vibration load on soil–water characteristic

#### Effect of vibration load on soil moisture content response

**Figure 4:**
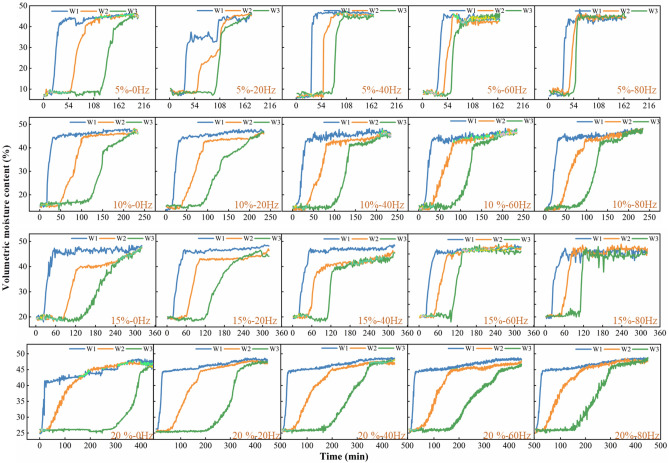
The temporal changes in the volumetric moisture content across various segments of the soil column.

The moisture content of soil columns is illustrated in Fig. 4, with emphasis on the influence of vibration frequency. The volumetric moisture content of three distinct soil layers (W1, W2, W3) showed different rate changes over time at various vibration frequencies (0 Hz, 20 Hz, 40 Hz, 60 Hz, 80 Hz) and initial moisture contents (5%, 10%, 15%, 20%). With increasing vibration frequency, the volumetric moisture content of all soil layers gradually rises during the seepage test. However, the rate at which the volumetric moisture content increases varies among soil layers and frequencies. At lower frequencies, the volumetric moisture content increases relatively slowly, while at higher frequencies, especially above 20 Hz, the volumetric moisture content increases more rapidly, leading to the early manifestation of a high volumetric moisture content state.

Various initial moisture levels significantly influence the dynamics of soil volumetric moisture content. With lower initial moisture content, e.g., 5% and 10%, the increase in volumetric moisture content is more noticeable, indicating increased sensitivity of dry soil to vibration. Conversely, with higher initial moisture content, approximately 15% and 20%, the soil volumetric moisture content tends to saturate and remains stable with increasing vibration frequency. These observations suggest that train vibration significantly accelerates the vertical migration and distribution of soil moisture, especially at higher vibration frequencies.

#### Effect of vibration load on PWP response

**Figure 5:**
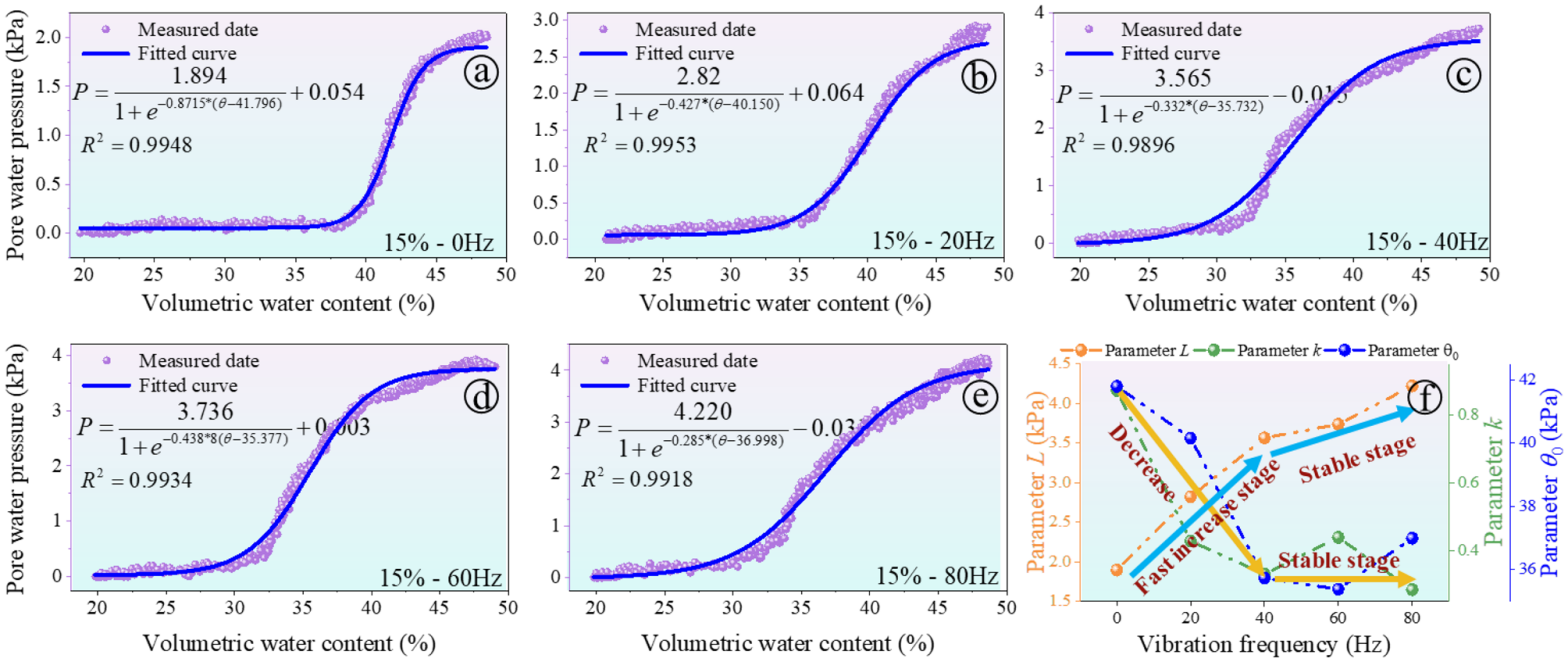
The influence results of the vibration on soil–water characteristic curve: (**a**) 0 Hz; (**b**) 20 Hz; (**c**) 40 Hz; (**d**) 60 Hz; (**e**) 80 Hz; (**f**) evolution of parameters.

Figure 5 illustrates the PWP response in a soil column with an initial moisture content of 15% exposed to varying vibration frequencies. This study measures the changes in pore water pressure along the sidewalls of the soil column, without measuring the matric suction within the soil itself. Results suggest that PWP initially increases non-linearly with rising moisture content, reaching a plateau afterward. This indicates the soil's heightened sensitivity to water within a specific moisture content range. However, beyond a critical value, the increase in water pressure amplitude diminishes. In Fig. 5a, without any vibration load, the soil's PWP stabilizes at 1.95 kPa. Conversely, under an 80 Hz vibration frequency, the peak PWP increases to 4.01 kPa, a 105% increase compared to the non-vibrating state. This suggests that higher vibration frequencies foster the development of soil pores and expedite water movement into these pores, resulting in a significant rise in PWP.

In this study, a sigmoid function was utilized to establish the relationship between unsaturated volumetric water content and pore water pressure (PWP), introducing vibration frequency as a variable. We developed a set of model parameters to capture the impact of vibration on the soil's hydraulic transmission properties.

2 $$PWP\left( {\theta ,f} \right) = \frac{L}{{1 + e^{{ - k\left( f \right)(\theta - \theta_{0} )}} }} + b$$

3 $$k\left( f \right) = k_{0} + k_{1} f$$

where *L* is a scale parameter represents the upper limit of the PWP, *L* is the upper limit value of the curve, *b* is the offset of the curve, *θ* is the function's offset parameter, shifting the curve up or down on the Y-axis. *θ*_0_ is the value of *w* at which the function's output is midway between its minimum and maximum. It can be thought of as an inflection point where the rate of increase in PWP begins to slow down, *k*_0_ is the base value of the slope adjustment factor when the vibration frequency is zero, *k*_1_ represents how much *k* (*f*) changes with each unit increase in the vibration frequency *f*, *f* is frequency of vibration.

Central to the model is the parameter *k*_*0*_, which represents the soil's inherent response to changes in PWP under static conditions (zero vibration frequency). This parameter is essential as it establishes the baseline hydraulic conductivity of the soil, serving as a reference point for understanding how vibrational forces alter soil behavior. *L* denotes the maximum PWP that the soil can sustain under different moisture levels, providing insight into the soil's capacity to handle pressure without reaching saturation. The parameter *θ*_*0*_ identifies the moisture content at which the rate of change in PWP begins to level off, offering a critical perspective on the soil's moisture retention capacity and its ability to transmit pressure.

The scattered points from Fig. 5a–e represent the measured data, and the accompanying curves show the fitted models. High coefficient of determination (R^2^) values, which exceed 0.995 for all cases, underscore the fit's accuracy. Notably, the fit results reveal a substantial influence of vibration frequency on soil hydraulic properties; frequencies above 20 Hz lead to the most significant changes. These changes are reflected in the dynamic parameters *L*, *θ*_*0*_, and *k*, especially beyond the 20 Hz threshold, which are pivotal in dictating both moisture transmission and retention within the soil matrix. The robustness of the model is validated by the consistently high R^2^ values across varying frequencies, confirming its efficacy in capturing the intricate relationship between pore water pressure and moisture content under different vibrational forces.

Results reveal that with an increase in frequency, *θ*_*0*_ and *k* exhibit an initial decrease followed by stabilization beyond 20 Hz (see Fig. 5f). This pattern suggests a decrease in the soil's sensitivity to moisture content changes, aligning with a stabilization of soil hydraulic transmission characteristics at higher frequencies. Similarly, the initial increase and subsequent stabilization of parameter L beyond 20 Hz reinforce the notion that the impact of vibration frequency on soil hydraulic properties diminishes at frequencies above 20 Hz.

### Effect of vibration load on soil hydraulic characteristics

#### Effect of vibration load on steady seepage time

**Figure 6:**
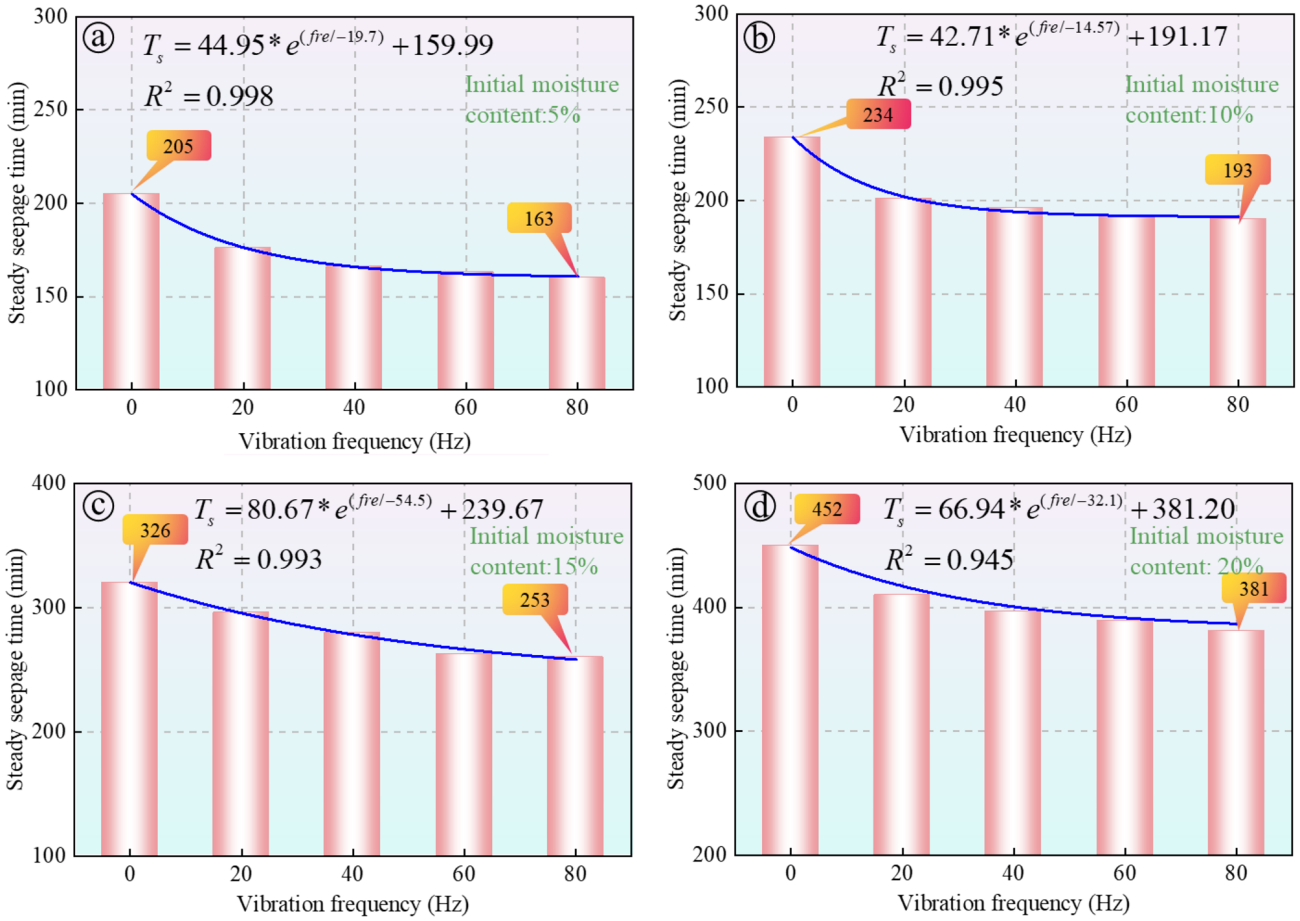
The impact of vibration on stable seepage time, the results are shown for different levels of initial moisture content: (**a**) 5%; (**b**) 10%; (**c**) 15%; and (**d**) 20%.

The study investigated the time required for soil to achieve stable seepage under various initial moisture contents (5%, 10%, 15%, and 20%) and at different vibration frequencies (0 Hz, 20 Hz, 40 Hz, 60 Hz, 80 Hz). Figure 6 presents the fitting analysis between stable seepage time and vibration frequency, precisely capturing the phenomenon where seepage behavior first rapidly changes and then stabilizes as vibration frequency increases, according to an exponential function relationship.

Fitting analysis indicates that stable seepage time decreases significantly with increasing vibration frequency, suggesting that higher frequency vibrations accelerate the degradation of soil structure. For example, in a soil sample with 5% moisture content, the stable seepage time decreased from about 205 min in the absence of vibration to approximately 163 min under 80 Hz vibration, a reduction of 20.4%. In a sample with 20% moisture content, seepage time decreased from 452 min without vibration to 381 min under 80 Hz vibration, a reduction of 15.7%. These findings demonstrate that train vibrations significantly impact soil hydraulic properties, with both vibration frequency and initial soil moisture content influencing soil stability.

The fitting results characterized the relationship between seepage time and vibration frequencies as a nonlinear downward trend, with fitting coefficients exceeding 0.98 in all cases, indicating a high level of fitting accuracy. The downward trend becomes less pronounced after exceeding a vibration frequency of 20 Hz, suggesting that the influence of vibration frequency on soil hydraulic properties is nonlinear and tends to stabilize beyond the optimal frequency of 20 Hz.

#### Effect of vibration load on the evolution of the wetting front

**Figure 7:**
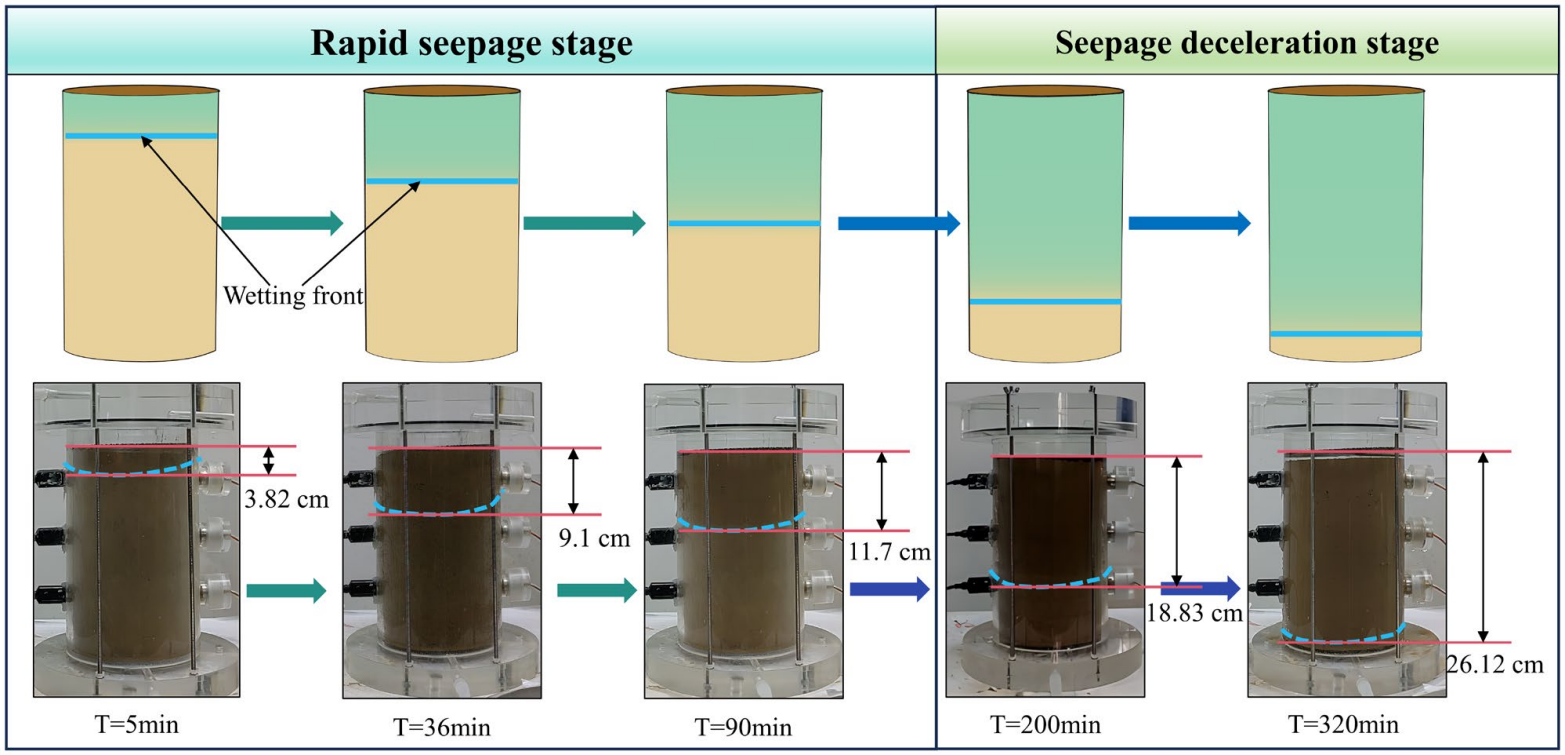
Hydraulic flow phenomena and morphology (15%-20 Hz).

Figure 7 illustrates the seepage process of a soil column with 15% moisture content when subjected to a 20 Hz vibration load. It is observed that the wetting peak (indicated by the blue dashed line) gradually moves downwards over time. The vibration load influences the infiltration and mobility of water by altering the arrangement and density of soil particles. Moreover, the non-linear increase in wetting depth with time suggests that the seepage process may be influenced by multiple factors, including the initial moistening state of the soil, soil texture, and porosity.

The movement of the wetting front in the soil column during the water seepage process can be divided into two stages: rapid seepage and seepage deceleration. The upper half of the figure uses different colors to distinguish the unsaturated area (brown) and the saturated area (cyan), while the lower half shows the progression over time. During the rapid penetration stage, water quickly penetrates the soil sample column, with the wetting front advancing to 3.82 cm in 5 min, 9.1 cm in 36 min, and 11.7 cm in 90 min. During this stage, most of the pores are filled with air, allowing water to pass through the pore network rapidly. In the infiltration deceleration stage, the wetting front reaches 18.83 cm at 200 min and 26.12 cm at 320 min. And most of the soil pores are filled with water, causing the infiltration rate to slow down. This observation highlights the significance of initial soil moisture content and pore structure as key factors influencing the rate of water penetration. Larger pore spaces facilitate rapid penetration in the early stage, but as saturation increases, the mobility of water and gas in the pores decreases, leading to a slower penetration rate.

**Figure 8:**
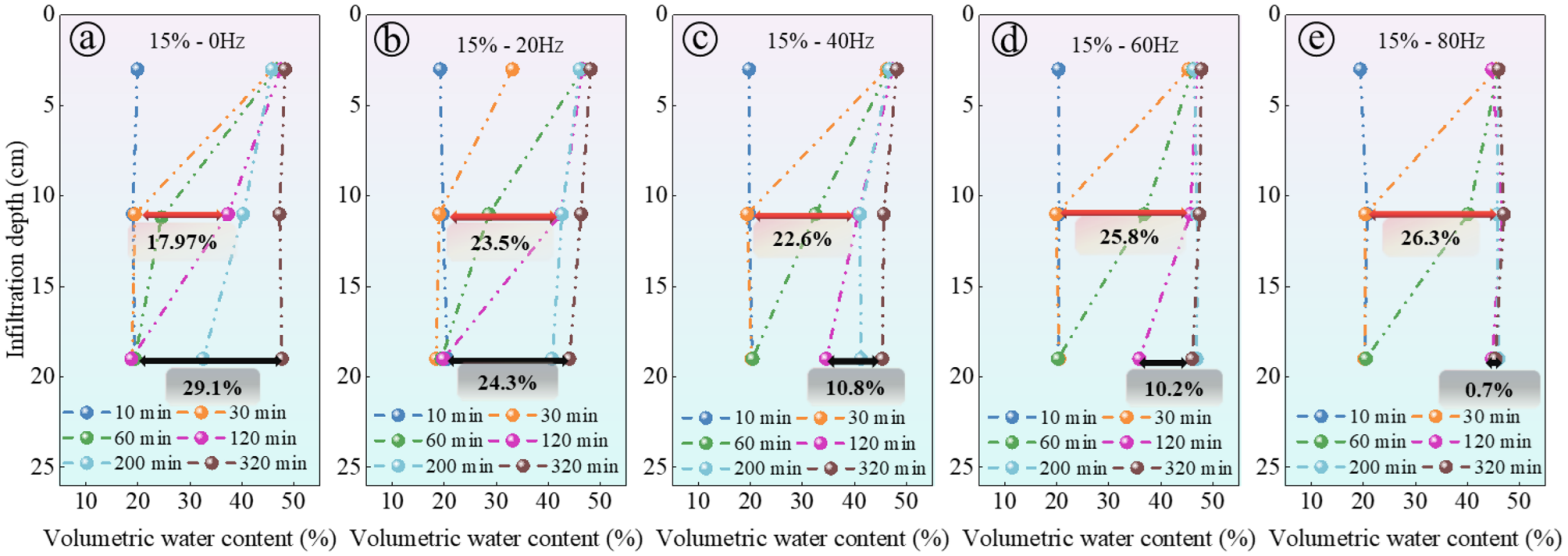
The progress of the wetting front in water infiltration at different frequencies: (**a**) 0 Hz; (**b**) 20 Hz; (**c**) 40 Hz; (**d**) 60 Hz; (**e**) 80 Hz.

Figure 8 illustrates the results, showing that as the vibration frequency increases, the difference in volumetric moisture content of the same soil layer at different time points also increases significantly. This change is indicated by a double-headed arrow and an adjacent percentage value in Fig. 8. The red double-headed arrow represents the change in volumetric moisture content at a depth of 11 cm in the soil column during the 30–120 min experiment, while the black double-headed arrow represents the change in soil volumetric moisture content at a depth of 19 cm when the experiment is carried out for 120–320 min. For instance, in Fig. 8a, under no vibration (0 Hz) conditions, the difference in volumetric moisture content at a depth of 11 cm is 17.97%, while under a vibration frequency of 80 Hz in Fig. 8e, the difference increases to 26.3%. On the other hand, the difference in volumetric moisture content at a depth of 19 cm decreases with the increase in vibration frequency, from 19.1% at 0 Hz to 0.7% at 80 Hz. That is to say, at higher vibration frequencies, the soil volumetric moisture content at the depth of 19 cm increases significantly after 200 min of testing. Thus, the increase in vibration frequency accelerates the migration rate of the wetting peak in the soil column, as high-frequency vibration promotes water movement in the soil.

#### Effect of vibration load on saturated hydraulic conductivity

**Figure 9:**
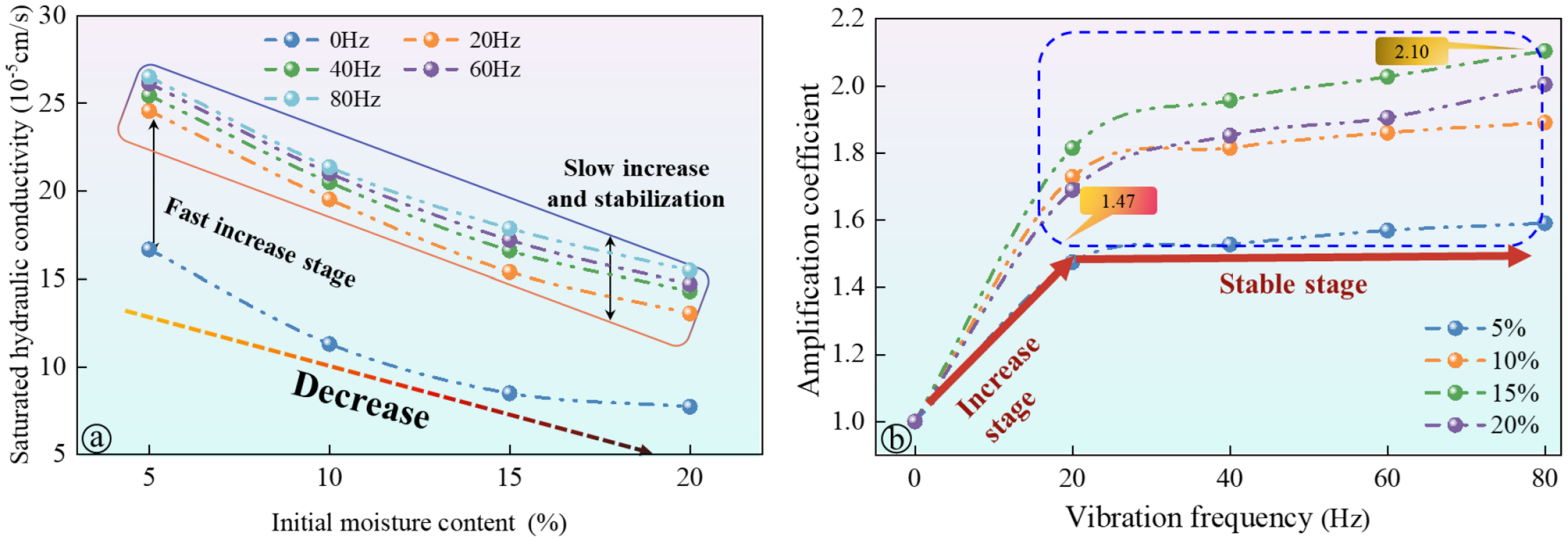
The influence results of the vibration on saturated hydraulic conductivity: (**a**) saturated hydraulic conductivity curve; (**b**) amplification coefficient curve of saturated hydraulic conductivity.

Figure 9a shows the relationship between soil saturated hydraulic conductivity and initial soil moisture content at different vibration frequencies (0 Hz, 20 Hz, 40 Hz, 60 Hz, and 80 Hz). The results indicate that as the frequency increases, the saturated hydraulic conductivity exhibits two stages: a rapid growth stage from 0 to 20 Hz, followed by a slower growth and stable stage beyond 20 Hz. In the low frequency range, the saturated hydraulic conductivity increases rapidly. However, as the frequency continues to increase, the growth rate of the coefficient slows down and stabilizes. For instance, when the soil column has a moisture content of 5%, the hydraulic conductivity increases from 16.67 × 10^–5^ cm/s at 0 Hz to 24.58 × 10^–5^ cm/s at 20 Hz, which represents a 47.4% increase. However, when the frequency is increased from 20 to 80 Hz, the hydraulic conductivity only increases by 7.8%. Additionally, an overall downward trend in the saturated hydraulic conductivity of the soil is observed with increasing initial soil moisture content.

To quantitatively assess the impact of train vibration loads on the saturated hydraulic conductivity of soil, this study proposes the use of an amplification factor, denoted as Ac, to represent the ratio of hydraulic conductivity under vibration conditions to that in the absence of vibration. The amplification factor is defined as the ratio of the saturated hydraulic conductivity measured at a specific vibration frequency (ksi) to that measured at zero vibration frequency (the static state, kso). This parameter was specially designed to capture the changes in conductivity due to vibration loads, reflecting alterations in soil structure and the distribution of pore water induced by vibration. The introduction of this parameter is based on observations of the relationship between vibration frequency and soil saturated hydraulic conductivity, taking into account that vibrations caused by passing trains can lead to changes in soil structure and pore distribution, thereby affecting the soil's hydraulic performance. This is shown in formula (4):

4 $$A_{c} = \frac{{k_{si} }}{{k_{s0} }}$$

where* A*_*c*_ represents the amplification factor of the saturated hydraulic conductivity. *k*_*si*_ refers to the saturated hydraulic conductivity at a vibration frequency of *i* Hz, while *k*_*so*_ represents the saturated hydraulic conductivity at a vibration frequency of 0 Hz.

The relationship between the amplification coefficient of soil saturated hydraulic conductivity and vibration frequency, considering different initial soil moisture contents (5%, 10%, 15%, and 20%) is shown in Fig. 9b. At low-frequency vibration, the amplification coefficients increase with frequency for all initial moisture contents, eventually reaching a stable stage after the vibration frequency surpasses 20 Hz. The initial moisture content significantly affects the stable value of the amplification coefficient of the saturated hydraulic conductivity. A higher initial moisture content corresponds to a larger stable amplification coefficient. For instance, at 20% moisture content, the stable value is approximately 2.10, while at 5% humidity, it is 1.47. This is because the saturated hydraulic conductivity of soil under high moisture content is low, and the structure changes under the action of vibration load. Compared with soil with low moisture content, the saturated hydraulic conductivity of soil with high moisture content has a greater room for increase.

### Effect of vibration load on soil pore microstructure

**Figure 10:**
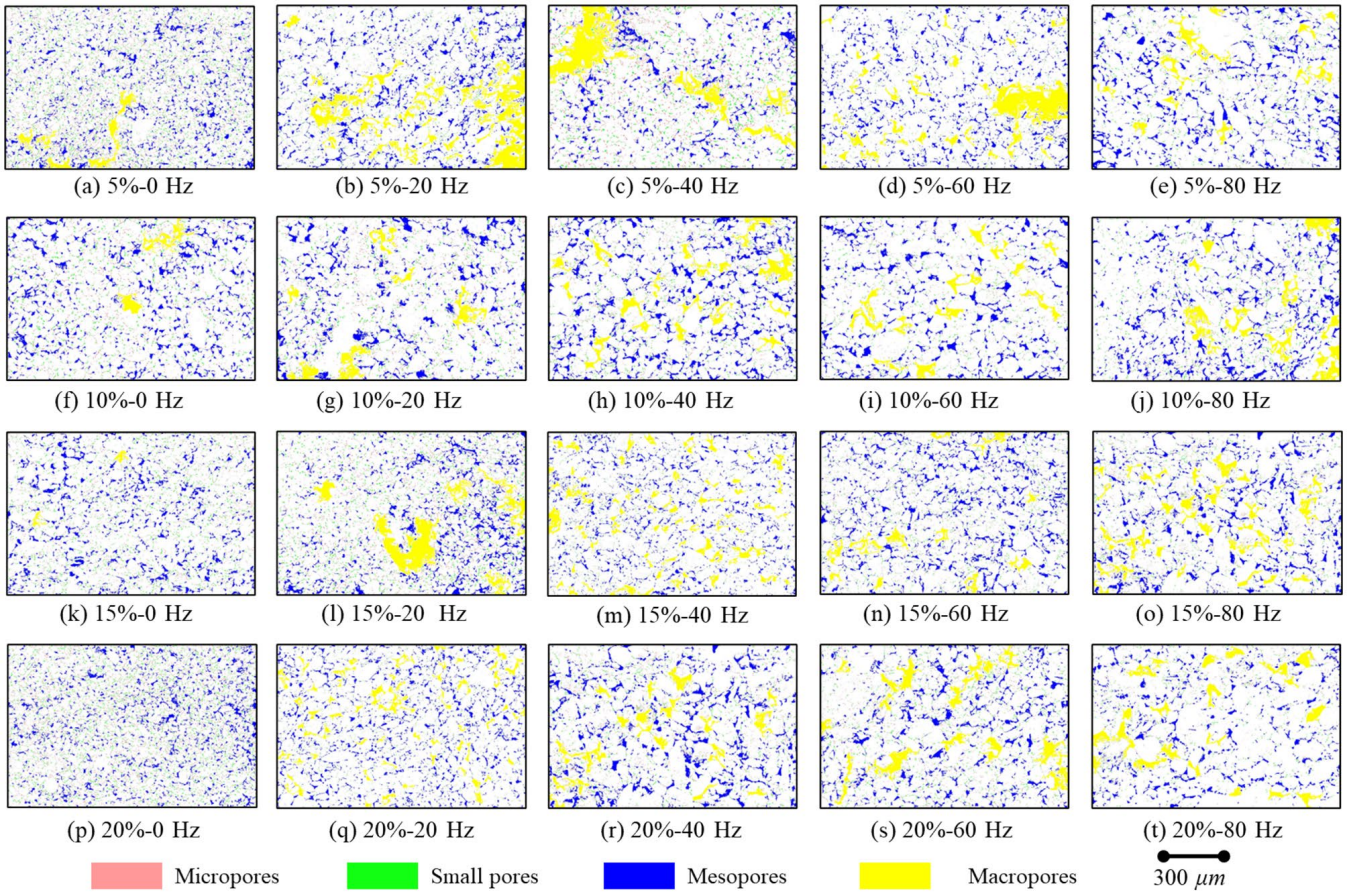
The results of the vibration's influence on the pore distribution of the soil samples.

Figure 10 presents the microstructure of soil samples with varying initial moisture contents (5%, 10%, 15%, 20%) and different vibration frequencies (0 Hz, 20 Hz, 40 Hz, 60 Hz, 80 Hz). Upon observing these images, it is evident that an increase in vibration frequency leads to a significant increase in the number and distribution of large pores (represented by the yellow area). This phenomenon can be attributed to the fact that high-frequency vibrations facilitate the redistribution of space between soil particles, resulting in the formation of larger pores. In the absence of vibration load (0 Hz), micropores and small pores are more scattered, whereas with vibration load (20 Hz to 80 Hz), macropores and mesopores become more pronounced. This suggests that vibrations may cause smaller pores to expand and develop into larger ones.

Changes in pore structure caused by vibration, particularly the increase in macropores, leads to changes in the pore structure of soil, enhancing its water penetration ability. The presence of larger pores allows water to flow at higher rates, resulting in an increase in the saturated hydraulic conductivity. The rearrangement and agglomeration of soil particles caused by vibration loading may contribute to the reduction in the number of micropores and small pores, promoting the formation of larger pores. As the frequency of vibration increases, the impact on soil pore structure becomes more pronounced. These changes in pore structure directly influence the hydraulic properties of the soil.

**Figure 11:**
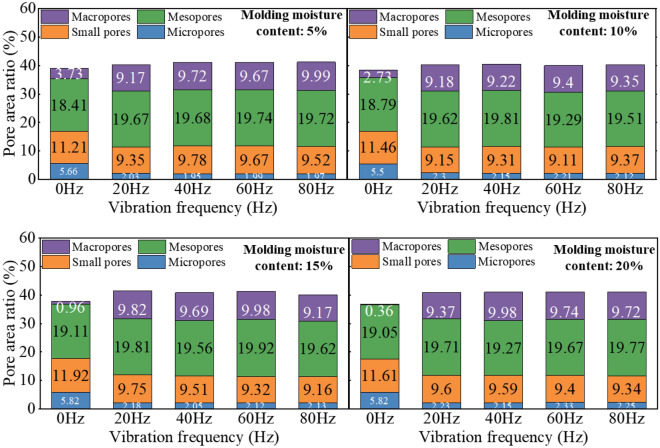
The effect of vibration on the proportion of pore area in soil samples with different moisture contents.

Figure 11 presents the alterations in soil pore size distribution when subjected to various vibration frequencies (0 Hz, 20 Hz, 40 Hz, 60 Hz, and 80 Hz) and different moisture contents (5%, 10%, 15%, 20%). The findings demonstrate that the proportion of large pores and small pores undergoes modifications with increasing vibration frequency. Specifically, as the frequency rises, the proportion of large pores escalates while the proportion of small pores diminishes.

**Figure 12:**
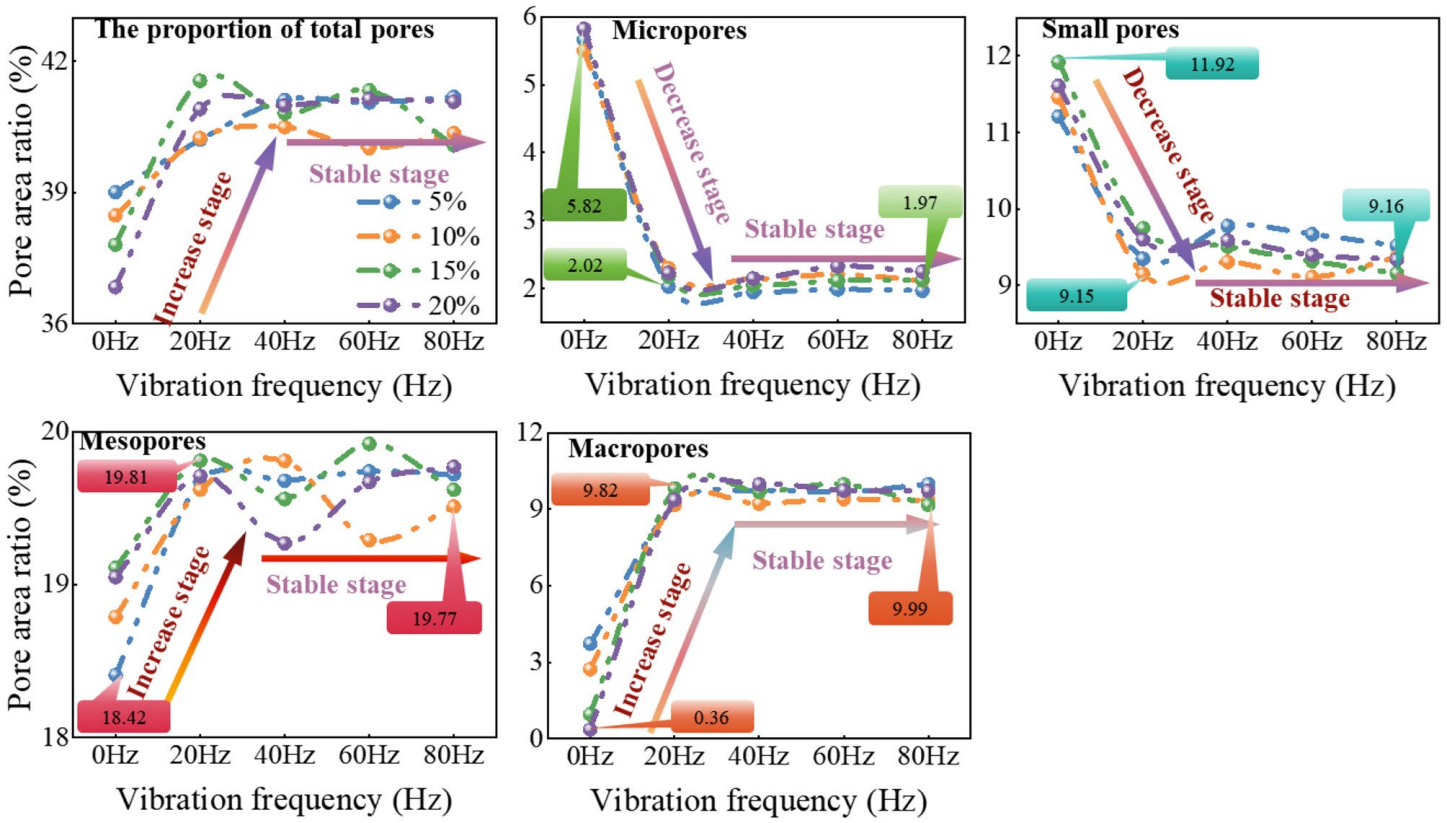
The influence of vibration on the proportion of different types of pores in the total area of soil sample images.

Figure 12 provides a detailed analysis of the relationship between the area ratio of different types of pores and vibration frequency. Initially, the total pore area ratio increases as the vibration frequency increases. However, once the vibration frequency exceeds 20 Hz, the total pore area ratio stabilizes. When considering pores with different diameters, the area ratio of micropores and small pores initially decreases with increasing vibration frequency before stabilizing. Specifically, the area ratio of micropores decreases from 5.82% at 0 Hz to 2.02% and then remains stable, while the area ratio of small holes decreases from 11.92% at 0 Hz to 9.15% and then stabilizes. On the other hand, the area ratio of mesopores and macropores initially increases with increasing vibration frequency before stabilizing. The area proportion of mesopores increases from 18.42% at 0 Hz to 19.81% and then stabilizes, while the area proportion of macropores increases from 0.36% at 0 Hz to 9.82% and then stabilizes. These findings highlight the significant impact of high-frequency train vibration on the soil pore structure, ultimately influencing the hydraulic properties of the soil.

**Figure 13:**
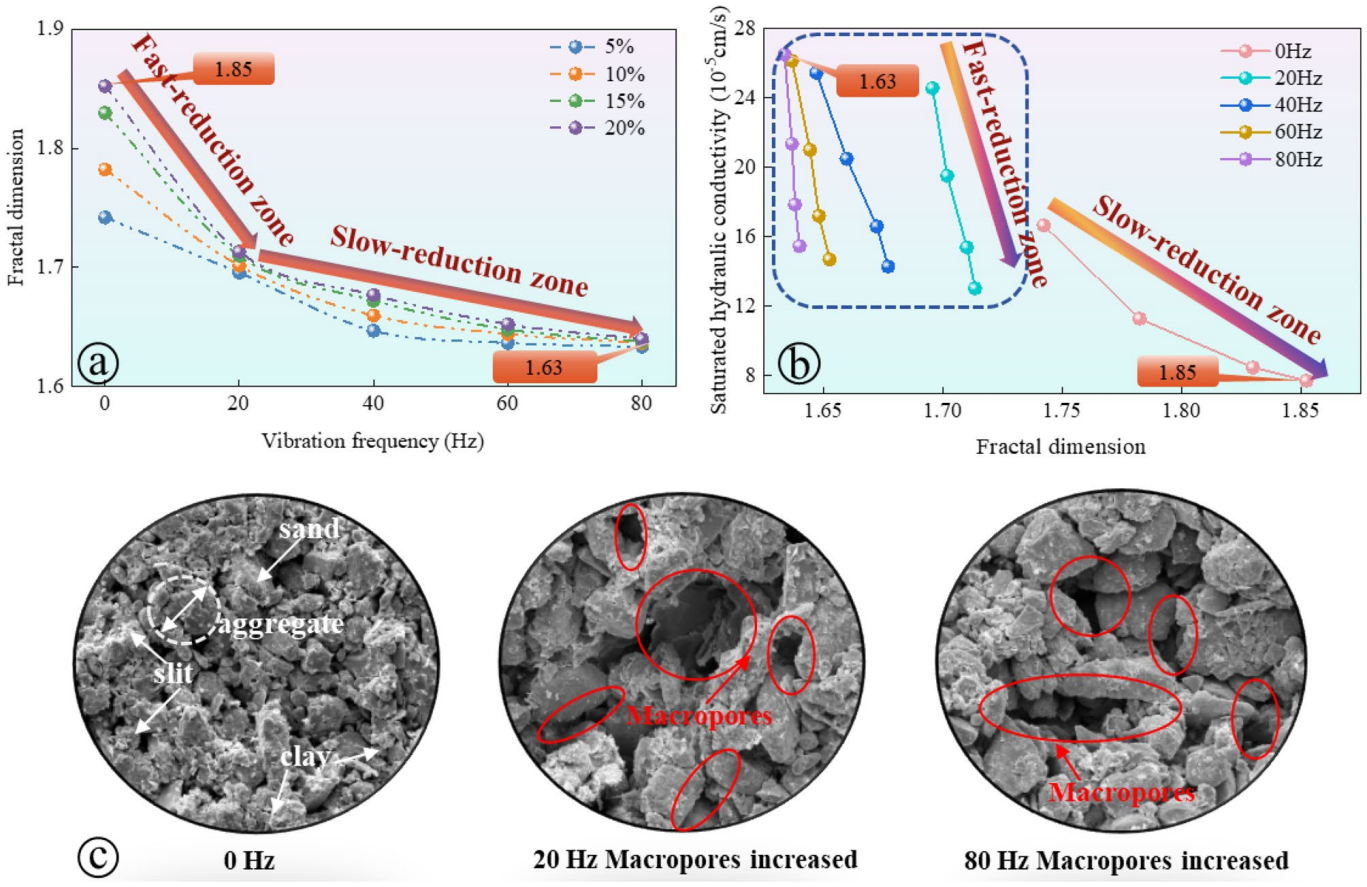
Microscopic characteristic parameters of loess pores after vibration at different frequencies: (**a**, **b**) results of vibration's impact on the fractal dimension of soil samples; (**c**) results of vibration's impact on the microstructure of soil samples.

The experiment considered various initial moisture contents (5%, 10%, 15%, and 20%) and different vibration frequencies (0 Hz, 20 Hz, 40 Hz, 60 Hz, 80 Hz). The evolution process of soil pore fractal dimension is illustrated in Fig. 13. Figure 13a illustrates the relationship between pore fractal dimension and vibration frequency at different initial moisture contents. As the vibration frequency increases, the fractal dimension generally decreases and can be divided into two regions: a 'rapidly decreasing area' and a 'slowly decreasing area'. In the low-frequency range (0–20 Hz), the fractal dimension decreases rapidly, indicating that the soil particles undergo quick rearrangement and the pore structure becomes more regular. As the frequency further increases (20–80 Hz), the rate of reduction in fractal dimension slows down, suggesting that the soil particles reach a dynamic equilibrium state and the changes in pore structure tend to stabilize. The fractal dimension serves as a parameter to quantify the complexity of soil pore structure, reflecting the irregularity and spatial heterogeneity of soil particle distribution. The decrease in pore fractal dimension signifies a decrease in pore complexity, which in turn promotes the conduction of water in soil pores.

Figure 13b illustrates the correlation between the saturated hydraulic conductivity and the fractal dimension of soil samples. The saturated hydraulic conductivity is a parameter that represents the soil's ability to facilitate water flow and is closely linked to the soil's pore structure. The figure demonstrates that a higher fractal dimension, indicating a more intricate soil pore structure, leads to a slower water flow and a lower soil permeability coefficient. Conversely, as the fractal dimension decreases, the complexity of the soil pore structure diminishes, resulting in improved pore connectivity and a significant enhancement in soil saturated hydraulic conductivity.

Figure 13c illustrates the changes in soil microstructure at different vibration frequencies (0 Hz, 20 Hz, and 80 Hz) using SEM images. The image highlights the components of sand, aggregate, silt, and clay, with the red circles indicating the enlarged pores caused by vibration. With an increase in vibration frequency, there is a noticeable rise in the number and size of macropores. This observation aligns with the decreasing fractal dimension and increasing hydraulic conductivity shown in Figures (a) and (b). The presence of these enlarged macropores facilitates greater hydraulic conductivity by providing wider pathways for water flow. These findings demonstrate that train vibration significantly impacts the soil's pore structure and hydraulic properties. The vibration load leads to a simplification of the soil structure, an increase in the number of macropores, and enhanced pore connectivity, ultimately improving hydraulic conductivity.

## Discussion

Previous studies have shown that soil microstructure plays a significant role in determining soil hydraulic properties^18,46^. For instance, Wang et al.^51^ conducted research on loess and found a strong correlation between changes in hydraulic conductivity and pore size distribution. However, these studies primarily focused on the link between soil microstructure and hydraulic properties, neglecting the impact of external loads on soil microstructure and hydraulic properties^52^. To address this gap, this study investigated the hydraulic properties of various soil groups under vibration loading. Our findings revealed a substantial improvement in the hydraulic conductivity of soil after applying vibration load (see Fig. 9). Notably, the soil's hydraulic conductivity exhibited a rapid increase within the low-frequency vibration range. However, as the frequency exceeded 20 Hz, the rate of increase in hydraulic conductivity slowed down. Additionally, our research demonstrated that vibration load significantly altered the microstructure of the soil (see Figs. 10, 11, 12), consequently enhancing its hydraulic conductivity (see Fig. 13). Conversely, the increased hydraulic conductivity also influenced the soil's microstructure. Previous studies highlight the reciprocal relationship between soil hydraulic properties and microstructure (e.g.,^8,53^). This study presents compelling evidence for the structural evolution of soils under long-term train vibrations. However, it is important to acknowledge certain limitations. The particle composition and gradation characteristics of soils vary across different areas, resulting in variations in their initial states such as structural strength, compaction, and pore morphology. Consequently, the response of soils to vibration loads and their subsequent evolution in terms of microstructure and hydraulic properties also differ significantly^54^. Therefore, future research will focus on evaluating the effects of vibration loading on the evolution of microstructure and hydraulic properties in different types of soils, considering their specific characteristics.

**Figure 14:**
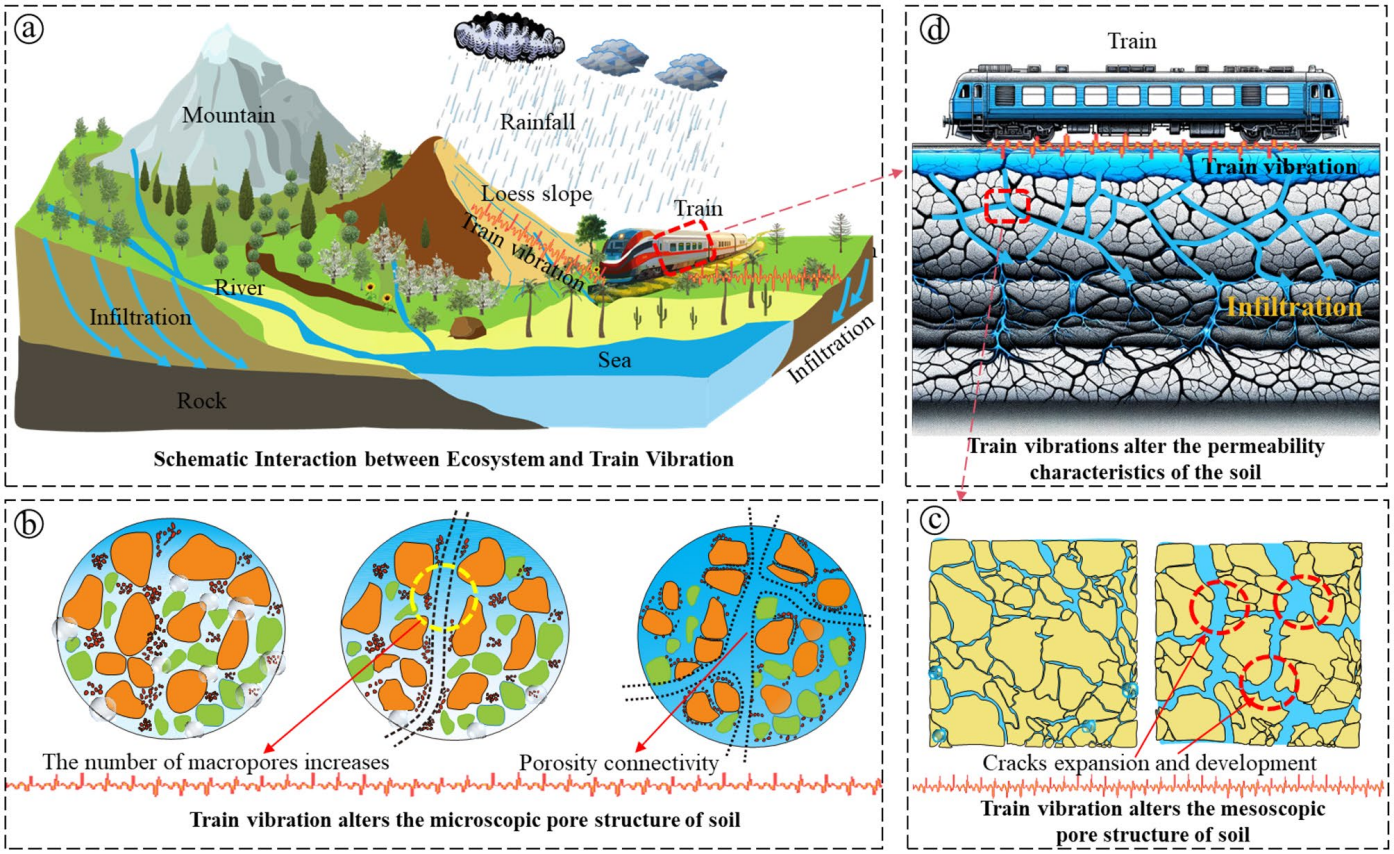
The influence mechanism of train vibration on soil hydraulic characteristics: (**a**) was modified from Xiao et al.^10^,(**b**–**d**) was drawn by the author.

Figure 14 illustrates the mechanism through which train vibration enhances water conduction in the soil. Figure 14a depicts a macroscopic diagram illustrating the impact of train vibration on the natural seepage process of surface water and groundwater^55,56^. The vibrations transmitted through the foundation can lead to the rearrangement of soil particles, thereby altering the soil structure and influencing water permeability^57^, 2019b).

The results in Section "Effect of vibration load on soil pore microstructure" indicate that vibration has a significant impact on the soil pore structure. It increases the number of mesopores and macropores, resulting in a simpler pore structure^58,59^. This, in turn, enhances the conduction rate of water in the soil. This process is illustrated in Fig. 14b and c. Figure 14b demonstrates the effect of vibration on the micropore structure of the soil. With continued vibration, the number of macropores increases, improving the connectivity between pores. This change in structure reduces resistance to water flow, enhances permeability, and accelerates water transport through the soil. Figure 14c depicts the influence of train vibration on the development and evolution of soil cracks at the mesoscopic scale. The expansion and development of cracks induced by vibration suggest a change in the microscopic structure of the soil, leading to the formation of new seepage channels^60^. These channels further facilitate the conduction of water in the soil.

Figure 14d demonstrates the impact of train vibration on soil hydraulic properties from a macro scale. It shows how train vibrations directly affect soil cracks and pores. Vibration not only causes the expansion of existing cracks but also creates new cracks, connecting them to form a crack network and increasing soil permeability^61^. The increased water mobility in the crack network accelerates water conduction in the soil, thereby affecting soil structure stability^62,63^. Figure 14 illustrates how train vibration affects soil pore structure and crack development characteristics from micro to macro scale. It highlights the reduction of micro-scale pore complexity and the development and connection of mesoscale cracks, which accelerate water conduction in the soil.

## Conclusion

1. The time required for the soil volumetric moisture content to reach its peak and stabilize rapidly decreases from no vibration (0 Hz) to a vibration frequency of 20 Hz. However, after the vibration frequency exceeds 20 Hz, the time required for the volumetric moisture content to reach its peak and stabilize slows down. Additionally, as the vibration frequency increases, the soil PWP also increases. For instance, at a vibration frequency of 80 Hz, the peak value of PWP increases by 105% compared to the non-vibration state. This indicates that higher vibration frequencies promote the development of soil pores and accelerate water migration.
2. As the vibration frequency increases, the stable seepage time of soil under different moisture content conditions significantly decreases by 15.7% to 20.4%. Additionally, the increased vibration frequency leads to a greater difference in soil volumetric moisture content at various depths, thereby promoting the migration rate of the wetting peak in the soil column. It is important to note that the soil hydraulic conductivity shows a rapid increase in the early stages of the vibration frequency increase, but its growth levels off after exceeding 20 Hz. This indicates that the effect of vibration on soil hydraulic properties is significantly enhanced in the frequency range from no vibration to 20 Hz, and after a further increase in frequency, this effect tends to stabilize.
3. As the vibration frequency increases, there is a significant change in the soil pore structure. The proportion of large pores increases while the proportion of small pores decreases. Micropores and small pores initially decrease and then stabilize. For instance, micropores decrease from 5.82% at 0 Hz to 2.02%, and small pores decrease from 11.92% to 9.15%. On the other hand, the area proportions of mesopores and macropores increase and then stabilize. Mesopores increase from 18.42% to 19.81%, and macropores increase from 0.36% to 9.82%. This demonstrates that under high-frequency vibration, soil pores transition from micropores and small pores to macropores and mesopores. This shift reduces pore complexity, accelerates the hydraulic conduction process, and influences soil hydraulic properties. It highlights the relationship between pore structure complexity and soil permeability.

## Informed consent

We have obtained explicit informed consent from all individuals participating in the study and/or their legal guardians, allowing the publication of identifiable information/images in an online open-access publication. The behavior of researchers collecting experimental data on site shown in Fig. 1 was completed by the first author of this article, Han Kai, and other researchers in the research group. We have taken all reasonable measures to ensure the privacy and personal identity information of research participants is protected, including but not limited to the removal of all patient names from text/figures/charts/images. The purpose of this statement is to ensure that our research complies with all relevant ethical and legal guidelines during the publication process, safeguarding the rights and privacy of research participants.

## Data Availability

All data generated or analyzed in this study will be provided upon request. For further inquiries, the corresponding author Wang Jiading can be directly contacted by email wangjd@nwu.edu.cn.
